# Supplementary material for: Global Syntactic Variation in Seven Languages: Toward a Computational Dialectology
Source: Front Artif Intell. 2019 Aug 14;2:15. doi: 10.3389/frai.2019.00015 (PMC7861279; doi:10.3389/frai.2019.00015)
Supplement: Supplementary file 1 [file Data_Sheet_1.pdf]

AR	Argentina	HN	Honduras	PT	Portugal
AU	Australia	HT	Haiti	PW	Palau
AZ	Azerbaijan	IE	Ireland	PY	Paraguay
BE	Belgium	IN	India	QA	Qatar
BF	Burkina Faso	IQ	Iraq	RE	Réunion
BG	Bulgaria	JO	Jordan	RU	Russia
BR	Brazil	KG	Kyrgyzstan	SI	Slovenia
BY	Belarus	KW	Kuwait	SN	Senegal
CA	Canada	KZ	Kazakhstan	SO	Somalia
CH	Switzerland	LT	Lithuania	SV	El Salvador
CL	Chile	LU	Luxembourg	SY	Syria
CM	Cameroon	LV	Latvia	TJ	Tajikistan
CO	Colombia	MD	Moldova	TN	Tunisia
CR	Costa Rica	MX	Mexico	UA	Ukraine
CU	Cuba	MY	Malaysia	UK	United Kingdom
CV	Cabo Verde	NC	New Caledonia	US	USA
DZ	Algeria	NG	Nigeria	UY	Uruguay
EC	Ecuador	NI	Nicaragua	UZ	Uzbekistan
EE	Estonia	NZ	New Zealand	VE	Venezuela
EG	Egypt	PA	Panama	ZA	South Africa
ES	Spain	PE	Peru		
FR	France	PF	French Polynesia		
GD	Grenada	PH	Philippines		
GE	Georgia	PK	Pakistan		
GT	Guatemala	PS	Palestine		

Table 1. Appendix A: Abbreviations for Countries

Arabic	Prec (CC)	Recall (CC)	F1 (CC)		Prec (TW)	Recall (TW)	F1 (TW)
AE	1.00	1.00	1.00	AE	–	–	–
DZ	–	–	–	DZ	0.98	0.98	0.98
EG	–	–	–	EG	0.99	0.99	0.99
IQ	–	–	–	IQ	0.97	0.97	0.97
JO	–	–	–	JO	0.97	0.97	0.97
KW	–	–	–	KW	0.94	0.96	0.95
PS	1.00	1.00	1.00	PS	–	–	–
QA	1.00	0.99	1.00	QA	–	–	–
RU	–	–	–	RU	1.00	1.00	1.00
SY	1.00	0.99	0.99	SY	0.97	0.97	0.97
**German**	**Prec (CC)**	**Recall (CC)**	**F1 (CC)**		**Prec (TW)**	**Recall (TW)**	**F1 (TW)**
AT	0.95	0.95	0.95	AT	0.96	0.91	0.93
CH	0.94	0.94	0.94	CH	–	–	–
DE	0.92	0.91	0.92	DE	0.95	0.98	0.97
LU	0.97	0.96	0.96	LU	–	–	–
PL	0.98	0.98	0.98	PL	–	–	–
**Portuguese**	**Prec (CC)**	**Recall (CC)**	**F1 (CC)**		**Prec (TW)**	**Recall (TW)**	**F1 (TW)**
AO	0.99	0.99	0.99	AO	–	–	–
BR	1.00	1.00	1.00	BR	1.00	1.00	1.00
CV	0.98	0.97	0.98	CV	–	–	–
PT	0.99	0.99	0.99	PT	1.00	0.99	1.00

Table 2. Appendix 2: Classification Performance for Arabic (Top), German (Middle), and Portuguese (Bottom), Web and Twitter Corpora with CxG-2 Features
